# Assessment of Fusion Gene Status in Sarcomas Using a Custom Made Fusion Gene Microarray

**DOI:** 10.1371/journal.pone.0070649

**Published:** 2013-08-13

**Authors:** Marthe Løvf, Gard O. S. Thomassen, Fredrik Mertens, Nuno Cerveira, Manuel R. Teixeira, Ragnhild A. Lothe, Rolf I. Skotheim

**Affiliations:** 1 Department of Cancer Prevention, Institute for Cancer Research, Norwegian Radium Hospital, Oslo University Hospital, Oslo, Norway; 2 Centre for Cancer Biomedicine, Faculty of Medicine, University of Oslo, Oslo, Norway; 3 Department of Biosciences, University of Oslo, Oslo, Norway; 4 Department of Clinical Genetics, Lund University Hospital, Lund, Sweden; 5 Department of Genetics, Portuguese Oncology Institute, Porto, Portugal; 6 Biomedical Sciences Institute (ICBAS), University of Porto, Porto, Portugal; CCR, National Cancer Institute, NIH, United States of America

## Abstract

Sarcomas are relatively rare malignancies and include a large number of histological subgroups. Based on morphology alone, the differential diagnoses of sarcoma subtypes can be challenging, but the identification of specific fusion genes aids correct diagnostication. The presence of individual fusion products are routinely investigated in Pathology labs. However, the methods used are time-consuming and based on prior knowledge about the expected fusion gene and often the most likely break-point. In this study, 16 sarcoma samples, representing seven different sarcoma subtypes with known fusion gene status from a diagnostic setting, were investigated using a fusion gene microarray. The microarray was designed to detect all possible exon-exon breakpoints between all known fusion genes in a single analysis. An automated scoring of the microarray data from the 38 known sarcoma-related fusion genes identified the correct fusion gene among the top-three hits in 11 of the samples. The analytical sensitivity may be further optimised, but we conclude that a sarcoma-fusion gene microarray is suitable as a time-saving screening tool to identify the majority of the correct fusion genes.

## Introduction

Fusion genes are found in haematological malignancies and soft tissue sarcomas [1] as well as in epithelial tumours [2], [3] and are known to be one of the most prominent types of genetic alteration in cancer [4]. The formation of a fusion gene leads to the presence of chimeric sequences, that can be used for diagnosis, prognosis or as targets for cancer treatment.

Sarcomas arise from bone and soft tissues, accounting for approximately 1% of all cancers in Norway [5] and can be classified into more than 40 subtypes [6]. These different subtypes show overlapping histological characteristics but still the classification has typically been based on histologic and immunophenotypic features. However, the identification of specific fusion genes in various subtypes allows correct classification of the tumours for the purpose of treatment and, in some cases, also provides prognostic information. Cytogenetic analyses, fluorescence in situ hybridization (FISH), reverse-transcription polymerase chain reaction (RT-PCR) or, in some cases, a combination of the methods are used to assess the correct fusion gene in the tumour. This assessment, however, can be cumbersome due to the promiscuity of fusion gene partners. For example, *EWSR1* is known to have multiple fusion gene partners in Ewing sarcoma (e.g., *FLI1*, *ERG*, *ETV1*, *FEV*, *ETV4*, and *PATZ1*). Furthermore, *EWSR1* is involved in several other sarcomas, but then with different partners, for instance extraskeletal myxoid chondrosarcoma (*NR4A3*), clear cell sarcoma (*ATF1*, *CREB1*), and myxoid liposarcoma (*DDIT3*) [7]. Therefore, to set the correct diagnosis it is not enough to show *EWSR1* rearrangement by for instance FISH, but multiple RT-PCR tests are also necessary.

Although the methods used today have their strengths, they also have weaknesses. Perhaps the largest drawback is the necessity to know which fusion gene(s) to expect beforehand and preferably also the most common breakpoint. We have previously published a custom designed fusion gene microarray [8] followed by a 2^nd^ improved version [9]. The microarray was designed to investigate all possible breakpoints between known individual fusion gene partners. In a previous study we investigated the fusion gene microarray's ability to detect fusion genes in cell lines from 15 different cancer types [9]. Here, we assess the fusion gene status by the microarray analysis of 16 clinical sarcoma samples, which were received from diagnostic labs.

## Materials and Methods

### Ethics statement

The study was carried out in accordance with the respective National legislations and with the Helsinki Declaration of 1975, as revised in 1983. The study was approved by the Regional Ethical Review Board of Lund University and the Institutional Review Board; Comissão de Ética para a Saúde. Written consent was given by all patients.

### Diagnostic samples

Sarcoma samples from diagnostic laboratories in Sweden (n = 6) and Portugal (n = 10) were included in the study (Table 1). The patients were diagnosed at Lund University Hospital between 1989 and 2009 and Porto Oncology Institute between 2001 and 2008, respectively. All samples were analyzed for the presence of fusion genes at their respective laboratories by karyotyping and/or RT-PCR [10]–[16] before this study started. The primer sequences and detailed laboratory protocols for detection of the individual fusion genes are given in the respective publications, all listed in Table 1. The particular assay for detection of *PAX7*-*FOXO1* is previously unpublished and uses QIAGEN OneStep RT-PCR Kit (QIAGEN, Inc., Valencia, CA) for fusion gene identification. Four µl primer mix (*PAX7F*: CCGACACCAGCTCTGCCTAC and *FOXO1R*: ATGAACTTGCTGTGTAGGGACAG) were mixed with 2 µl RT-Taq mix, 10 µl 5× Reaction mix, 10 µl 5× Q-solution, 2 µl dNTP mix, 1 µg RNA, and water to a final volume of 50 µl. The PCR reaction was then incubated at 50°C for 30 minutes and 95°C for 15 minutes before cycled 35 times through 95°C for 30 seconds, 60°C for 1 minute and 72°C for 1 minute. The incubation was completed with 7 minutes at 72°C.

**Table 1:** Samples investigated in the study.

Sample	Origin	Fusion gene	Diagnosis	Rank^1^	Ref^2^
3065	Portugal	*SS18-SSX2*	Synovial sarcoma	1	[10]
6499	Portugal	*SS18-SSX2*	Synovial sarcoma	1	[10]
7757	Portugal	*SS18-SSX2*	Synovial sarcoma	1	[10]
9972	Portugal	*SS18-SSX1*	Synovial sarcoma	1	[10]
2430/90	Sweden	*SS18-SSX1*	Synovial sarcoma	1	[10]
14319	Portugal	*PAX7-FOXO1*	Alveolar rhabdomyosarcoma	1	In-house
168/97	Sweden	*EWSR1-NR4A3*	Extraskeletal myxoid chondrosarcoma	1	[11]
3490/00	Sweden	*FUS*-*CREB3L2*	Low grade fibromyxoid sarcoma	1	[12]
244/09	Sweden	*FUS*-*CREB3L2*	Low grade fibromyxoid sarcoma	2	[12]
885/89	Sweden	*FUS*-*DDIT3*	Myxoid liposarcoma	2	[14]
10908	Portugal	*EWSR1-FLI1*	Ewing sarcoma	3	[16]
14476	Portugal	*EWSR1-FLI1*	Ewing sarcoma	7	[16]
4640	Portugal	*EWSR1-FLI1*	Ewing sarcoma	9	[16]
2492/08	Sweden	*EWSR1*-*FLI1*	Ewing sarcoma	24	[15]
10408	Portugal	*FUS*-*DDIT3*	Myxoid liposarcoma	28	[13]
3214	Portugal		Fibrosarcoma		NK

1 All 38 investigated fusion genes are ranked based on the likelihood of being the correct fusion gene in the particular sample. Lower numbers indicate higher likelihood.

2 Information about RT-PCR protocols for fusion gene detection. In house: protocol not previously published. See Materials and Methods for more details. NK: Normal karyotype.

Fifteen samples diagnosed with known sarcoma-related fusion genes were included alongside with one sample without.

### Microarray design and laboratory protocol

The oligo design and laboratory procedures were followed according to our previously described protocol [9]. In short, we compiled a database of 548 reported fusion genes. This contained all records in the major public fusion gene databases [4], [17], [18] in addition to fusion genes reported from deep sequencing studies and those found in a thorough literature search. We then designed oligo probes targeting every exon in all of the fusion gene partners, in addition to chimeric exon-exon probes that target every possible exon-exon combination in fusion gene pairs. The total set of 599,839 oligos was synthesized onto 3×720k HD2 microarrays (Roche NimbleGen, Inc., Madison, WI).

Total RNA from the Swedish sarcoma samples was isolated using the Trizol reagent (Life Technologies, Inc. Rockville, MD) according to the producers' protocol. Portuguese samples were immersed in RNAlater (Ambion Inc, Austin, TX) after excision, for inactivation of RNases and RNA stabilization, and frozen at −80°C until RNA extraction. Total cellular RNA was extracted from 250 mg of tumour tissue using the FastRNA Kit Green (Qbiogene, Carlsbad, CA) for 45 seconds, with a speed rating of 6.0 in a FastPrep FP120 instrument (Qbiogene). RNA was quantified spectrophotometrically at 260 nm and stored at −80°C. RNA quality was evaluated, for all samples, by use of the Agilent 2100 Bioanalyzer (Agilent Technologies, Palo Alto, CA). All samples had RNA integrity numbers (RIN)>5. Ribosomal RNA was removed and double stranded cDNA made from the remaining RNA using random hexamer primers. The double stranded cDNA was then labelled with Cy3 and hybridized onto the fusion gene microarray for 16–20 hours, before washed, dried and scanned. All procedures and reagents with corresponding manufacturer have previously been described in detail [9].

### Data processing and analysis

Only fusion genes known from sarcomas were included in the data analysis (Table S1). The fusion scoring algorithm is built on the bioinformatic analysis of the expression intensity of the chimeric oligos combined with the intragenic expression profiles of fusion partners (gene *A* and *B*) from our previous publication [9]. We have here further developed this algorithm by adding two steps, one to avoid what is referred to as half-binder effects and one to enhance strong unique chimeric probe scores. Further, we made a minor change to the expression profile scoring part of the algorithm to allow atypical expression profiles. The resulting automatic scoring profiles are a result of these computer based improvements which are explained in more detail below.

The half-binder reduction step is necessary when a part of a chimeric probe has such strong binding affinity that it shows strong signals even though only part A or part B of the probe A+B binds to cDNA in the sample. This phenomenon and the effect of this filter are seen in Figure S1. To reduce such half-binder noise we iterate all rows and columns to rank all scores on that row/column. If 25% or more of the probes within one row or column show an expression level equal to or above 70% of the maximum score in the row or column, or three or more probes have a score equal to or above the maximum chimeric cutoff (3.0), all values in the particular row or column are set to 0. All non-adjusted rows and columns remaining unfiltered but where 25% or more of the probes show an expression level equal to or above 50% of the maximum score in the row or column are tagged with “weak half-binding present”.

The enhancement of individual strong chimeric probes was implemented to ensure weight for those which are noticeable as outlier values as compared to the rest of the chimeric oligos. These may previously have been underestimated as compared to the intragenic oligos. We have observed that true positives often show a heat map signature with one significantly warmer probe than all others, or a tight cluster of highly expressed probes indicating possible fusion gene transcript variants. The enhancement step was implemented by iterating all chimeric probe values (after removal of the half-binder effects) for each possible fusion transcript. If the maximum chimeric value was 0.8, or more, larger than the second largest chimeric value in the heat map, the value was adjusted to 6.0 (3.0 if the row or column of the maximum chimeric probe value within the heat map was already tagged with “weak half-binding effect present”).

The parameters used to define whether to perform reduction of half binder effects or single probe enhancement were set after an iterative optimization process including interpretation of plots of score distributions for true and false positives.

The automated fusion scoring algorithm is described below in pseudocode, where updates to the previous version are underlined. The fusion partner genes *A* and *B* are here defined by *A_1_*+*A_2_* = *A* and *B_1_*+*B_2_* = *B*, where the fusion product will be *A_1_*+*B_2_* and/or *B_1_*+*A_2_*. Our fusion gene microarray only probes for the A_1_+B_2_ constellation. In the following pseudocode, “len(*X_n_*)” describes the number of exons included in the *n* ^th^ part of gene *X* and *X_n_* is the average expression level of the exons included in part *X_n_* of gene *X*. The final fusion score is now computed similarly for all possible *A_n_*+*B_n_* expression profiles as opposed to the previous algorithm that only allowed (*A_1_*>*A_2_* and *B_1_*<*B_2_*) or *vice versa* and had a special scoring function for each case.

def score(A_1_, A_2_, B_1_, B_2_, chimeric_cutoff = 3.0, max_break_score = 1.5, adjusted_to_max = 6.0)

if len(*B_1_*) =  = 0:

 fusion_score = 0.1

else if (*A_1_*>*A_2_* and *B_2_*<*B_1_* and len(*A_2_*)≤1 and len(*B_2_*)≤1) or

(*A_1_*<*A_2_* and *B_2_*>*B_1_* and len(*A_2_*)≤1 and len(*B_2_*)≤1):

fusion_score = 0.1

else if *A_1_*<*A_2_* and *B_2_*>*B_1_* and len(*A_1_*)≤1 and len(*B_1_*)≤1:

fusion score = 0.1

else if (*A_1_*>*A_2_* and *B_1_*<*B_2_* and len(*B_2_*)≤1) or

 (*A_1_*<*A_2_* and *B_1_*>*B_2_* and len(*A_2_*)≤1):

 fusion_score = 0.1

else:

 if chimeric_score(*A*_1_+*B*_2_) =  = adjusted_to_max ##handles “single-strong-probe enhancement”

 fusion_score = chimeric_score(*A*_1_+*B*_2_)

else:

 fusion_score = min(chimeric_score(*A*_1_+*B*_2_), chimeric_cutoff)

 break_score_A = min(abs(*A_2_*−*A_1_*), max_break_score)

 break_score_B = min(abs(*B_2_*−*B_1_*), max_break_score)

 fusion_score = fusion_score+break_score_A+break_score_B

return fusion_score ## The total score of this possible chimeric break-point (maximum 6.0).

Raw and processed data were deposited to the Gene Expression Omnibus public repository for microarray data (accession number GSE43632) according to the MIAME, minimum information about a microarray experiment, recommendations for recording and reporting microarray-based gene expression data [19].

## Results

Sixteen sarcoma samples initially analysed in diagnostics labs by chromosomal G-banding, FISH and RT-PCR to identify the particular disease related fusion genes were here analysed by a fusion gene microarray. One sample without a known fusion gene and fifteen samples with various known fusion genes were included

For each of the 16 sarcoma samples, an automated scoring algorithm set a fusion score to each of the 38 sarcoma-related fusion genes on the microarray. The fusion score was calculated by a combination of results from the chimeric oligos and results from oligos targeting sequences within all exons of each partner gene, and their differences before and after each possible breakpoint within all known transcript variants (see Data processing and analysis above).

**Figure 1:**
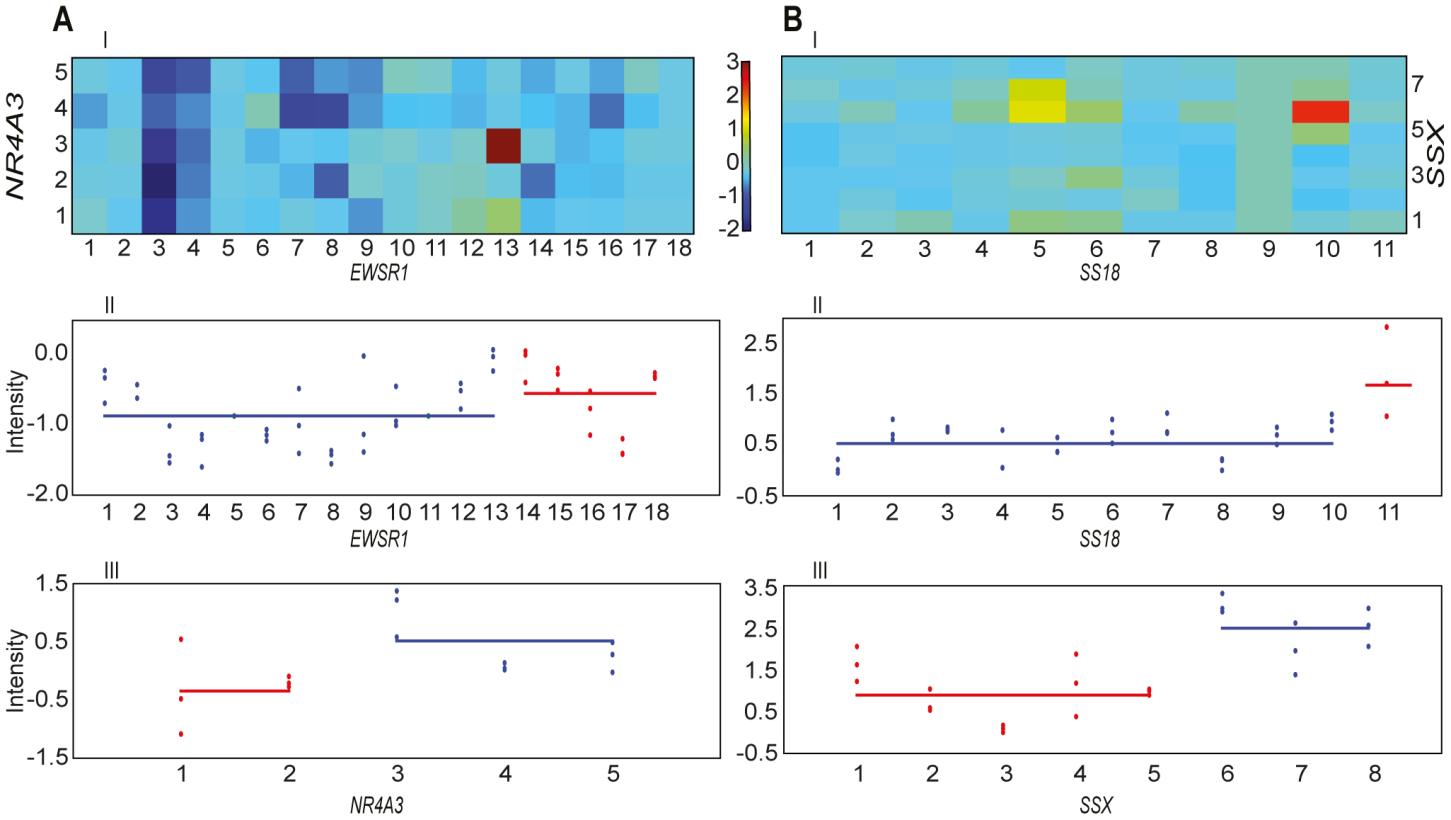
Top ranked fusion genes in two sarcoma samples. A. *EWSR1*-*NR4A3* in the 168/97 sample. B. *SS18*-*SSX1* in the 9972 sample. I) Intensity heat map of chimeric oligos. Each square represents one possible exon-exon boundary between the two gene partners. One square is highly expressed (A: 13-3, B: 10-6) and reflects the presence of chimeric RNA covering the corresponding exon-exon boundary. This fusion breakpoint corresponds with a shift in relative expression measured by intragenic oligos covering both the upstream (II) and downstream (III) fusion gene partners. Blue and red colours represent the two possible chimeric transcripts generated from the fusion gene.

In eight of the 15 samples with a known fusion gene, the automated scoring of the microarray data ranked the correct fusion gene as number one (Table 1 and exemplified in Figure 1). Furthermore, the correct fusion gene ranked among the top three hits in 11 of the 15 samples. It was not possible to separate the sample with no known fusion gene from the samples where the correct fusions were not found.

The altogether 16 analyzed samples represent seven different sarcoma diagnoses (Table 1), three of which (synovial sarcoma, alveolar rhabdomyosarcoma, and extraskeletal myxoid chondrosarcoma) could be correctly identified using only the fusion gene microarray. For the two samples of low grade fibromyxoid sarcoma, one could be identified correctly while the other one had the correct fusion gene ranked second. For the remaining two diagnoses (Ewing sarcoma and myxoid liposarcoma) the correct fusion genes were not identified.

## Discussion

We have in the present study shown that the fusion gene microarray can be used to detect fusion genes in clinical diagnostic sarcoma samples without any previous knowledge about which of the sarcoma fusion genes to expect. In addition to identifying the particular fusion gene, the microarray is designed to locate the exact breakpoint between the two fusion gene partners. These are important improvements compared to the standard methods used in clinical diagnostics laboratories today. For instance, with both FISH and RT-PCR, only one fusion gene or one fusion partner, is tested for per analysis, implying that the correct fusion gene will only be found if the analyst is able to upfront choose the correct fusion gene assays for each particular sample. In reality, this means that multiple assays are needed to locate the correct fusion gene and the breakpoints of both partners. For example, a break-apart FISH probe can be used to assess whether or not the *EWSR1* gene is rearranged. Such a probe will be split in two if the *EWSR1* gene is rearranged. However, it cannot say anything about the fusion partner of *EWSR1*. *EWSR1* has at least 13 different partners in sarcomas (Table S1), and, furthermore, for some known fusion genes the breakpoints vary considerably. Consequently, it is difficult to test for all possible fusion transcripts using traditional RT-PCR. While FISH with break-apart probes readily identifies the vast majority of *EWSR1* rearrangements, it does not provide any information on the fusion partner. In addition, there are no commercial probes for many of the genes involved in sarcoma-associated fusions. In this context, the fusion gene microarray described here might be very useful; in a single assay, using amounts of RNA that should be possible to extract from both tumour biopsies and needle aspirates, all known sarcoma-associated fusion genes can be tested for.

Thus, for the >50% of sarcoma samples for which the fusion gene microarray pinpointed the correct fusion gene as the top hit, a substantial work-saving can be accomplished as the complex fusion gene detection pipe-line which is currently in routine use can be reduced to simple validation of the top hit by RT-PCR. However, the remaining half of the samples where the correct fusion gene was not picked up, represent the subset of sarcomas for which the current version of the fusion gene microarray cannot replace the existing methodology. However, the fusion gene microarray gives clear suggestions on where to continue the search by indicating the most likely fusion gene candidates and their breakpoints. Focusing on these particular breakpoints with RT-PCR could save both time and work effort. The suboptimal detection of all fusion genes can both be due to low expression or suboptimal probe design for the true positive fusion genes.

Included in the present study were sarcomas being correctly classified with fusion genes such as *PAX7-FOXO1*, *EWSR1-NR4A3*, *FUS-CREB3L2*, and *SS18-SSX*. In five of the investigated samples *SS18* was fused to an *SSX* gene. The *SSX* homologues show very high sequence identity, and the three known SS18-SSX fusion genes are therefore difficult to separate from each other using the fusion gene microarray. However, as they are all specific for synovial sarcoma, and the different fusion forms do not have any prognostic implications, the fusion gene microarray will still provide clinically important information.

In a recent publication, another approach using custom oligo microarrays for combined targeting of chimeric breakpoints and intragenic breakpoints was described [20]. Here, the oligo design strategy was similar to the one first described by us [8], [9], but they employed an antibody based detection of RNA hybridised to DNA oligo sequences on the array. This publication included results from sarcoma samples analysed on a pilot array including only two fusion genes, *EWSR1-FLI* and *EWSRI-ERG*.

Our fusion gene microarray has been designed to detect all known fusion genes in one single experiment, and the database version applied for the current design included 548 fusion genes. However, since all the samples used for the present study were sarcomas, the bioinformatics analysis was restricted to the 38 fusion genes known from sarcomas. Upon implementation of the fusion gene microarray in a routine clinical laboratory, it would be natural to create a specific chip including only the fusion genes needed to distinguish between the differential diagnostic possibilities (e.g. all fusion genes known from sarcomas and lymphomas). The necessarily smaller number of features would make it possible to optimise the particular probe sequences for these fusion genes even further. Here, even manual design would be feasible of particular challenging chimeric junction sequences and intra-genic sequences with homology between different fusion partners. The latter would, for example, enable separation of gene partners such as *SSX1* and *SSX2*. As Table 1 shows, 13 of the 15 samples with known fusion gene has the correct fusion gene ranking among the top-nine hits, indicating the great potential for improving the percentage of correct diagnoses in a specialised sarcoma-diagnoses chip. In addition, the smaller number of features would make it possible to have multiple arrays, and thus multiple samples to be tested, per microarray, reducing both costs and time consume per sample/tumour.

The growing field of high-throughput sequencing, with detection of both known and novel fusion genes, shows great potential for implementation in diagnostics. However, it is still necessary to reduce both costs and amounts of intricate analyses before the method can be used routinely for this purpose. In comparison, a specific fusion gene chip, designed to distinguish between the different sarcoma subtypes, would have benefits like low costs, low time consume due to multiple samples per array, and small amounts of follow-up analyses. Furthermore, the data storage, handling and analysis can be done on a standard personal computer, and no large-scale hardware infrastructure is needed, as for high-throughput sequencing facilities.

In conclusion, we have investigated 16 clinical diagnostic sarcoma samples by the fusion gene microarray technology, and demonstrated that this can be used to identify the correct fusion gene in more than half of the cases. This can potentially speed up the molecular diagnostics of sarcomas, and also save resources from the current time-consuming and complex fusion gene detection pipeline which can be reduced to RT-PCR confirmation where positive fusions are found by the microarray.

## Supporting Information

Figure S1 **Effect of additional filters.** Effect of additional filters added to the previously published algorithm (sample 3065). A) Heat map of chimeric probes for a false positive fusion gene before (I) and after (II) correction for striping (resulting from instances of unspecific oligo half-binding). Before correction for striping, the wrong fusion gene (*SSX-SS18L1*) was ranked first. Removal of striping reduced the fusion score of this fusion gene and the new plot (II) is no longer ranked first. B) Heat map for a false negative fusion gene. This correct fusion gene, *SSX-SS18*, was ranked second before addition of filters (I), but ranked first after addition of the “single-strong-chimeric-probe” filter (II). Removal of striping helps decrease the number of false positives and the “single-strong-chimeric-probe” filter helps enhance true positive fusion genes.
(PDF)

Table S1 **Fusion genes known from sarcomas.**
(DOCX)
