# Supplementary material for: Assessment of Fusion Gene Status in Sarcomas Using a Custom Made Fusion Gene Microarray
Source: PLoS One. 2013 Aug 13;8(8):e70649. doi: 10.1371/journal.pone.0070649 (PMC3742753; doi:10.1371/journal.pone.0070649)
Supplement: Figure S1 — Effect of additional filters. Effect of additional filters added to the previously published algorithm (sample 3065). A) Heat map of chimeric probes for a false positive fusion gene before (I) and after (II) correction for striping (resulting from instances of unspecific oligo half-binding). Before correction for striping, the wrong fusion gene (SSX-SS18L1) was ranked first. Removal of striping reduced the fusion score of this fusion gene and the new plot (II) is no longer ranked first. B) Heat map for a false negative fusion gene. This correct fusion gene, SSX-SS18, was ranked second before addition of filters (I), but ranked first after addition of the “single-strong-chimeric-probe” filter (II). Removal of striping helps decrease the number of false positives and the “single-strong-chimeric-probe” filter helps enhance true positive fusion genes. (PDF) [file pone.0070649.s001.pdf]

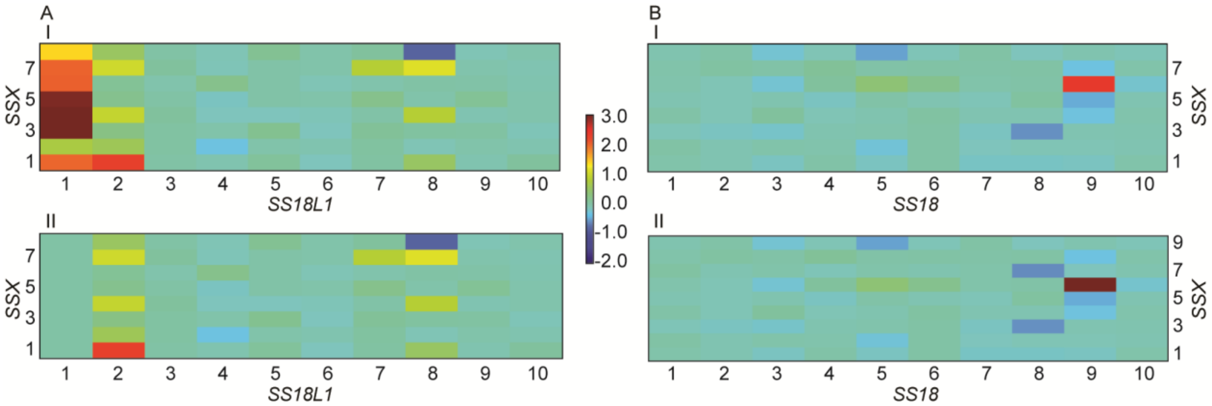

Figure S1. Effect of additional filters

Effect of additional filters added to the previously published algorithm (sample 3065). A) Heat map of chimeric probes for a false positive fusion gene before (I) and after (II) correction for striping (resulting from instances of unspecific oligo half-binding). Before correction for striping, the wrong fusion gene (*SSX-SS18L1)* was ranked first. Removal of striping reduced the fusion score of this fusion gene and the new plot (II) is no longer ranked first. B) Heat map for a false negative fusion gene. This correct fusion gene, *SSX-SS18*, was ranked second before addition of filters (I), but ranked first after addition of the "single-strong-chimeric-probe" filter (II). Removal of striping helps decrease the number of false positives and the "single-strong-chimeric-probe" filter helps enhance true positive fusion genes.
