# Supplementary material for: Assessment of Fusion Gene Status in Sarcomas Using a Custom Made Fusion Gene Microarray
Source: PLoS One. 2013 Aug 13;8(8):e70649. doi: 10.1371/journal.pone.0070649 (PMC3742753; doi:10.1371/journal.pone.0070649)
Supplement: Table S1 — Fusion genes known from sarcomas. (DOCX) [file pone.0070649.s002.docx]

### Table S1. Fusion genes known from sarcomas

| 5' gene (HGNC symbol) | 5' gene (Ensembl) | 3' gene (HGNC symbol) | 3' gene (Ensembl) | Sarcoma type |
| --- | --- | --- | --- | --- |
| *ASPSCR1* | ENSG00000169696 | *TFE3* | ENSG00000068323 | Alveolar soft part sarcoma |
| *CIC* | ENSG00000079432 | *DUX4* | ENSG00000212638 | Primitive undifferentiated soft tissue sarcoma |
| *COL1A1* | ENSG00000108821 | *PDGFB* | ENSG00000100311 | Dermatofibrosarcoma protuberans |
| *EPC1* | ENSG00000120616 | *PHF1* | ENSG00000112511 | Endometrial stromal sarcoma |
| *ETV6* | ENSG00000139083 | *NTRK3* | ENSG00000140538 | Infantile fibrosarcoma |
| *EWSR1* | ENSG00000182944 | *ATF1* | ENSG00000123268 | Clear cell sarcoma, Angiomatoid fibrous histocytoma |
| *EWSR1* | ENSG00000182944 | *CREB1* | ENSG00000118260 | Clear cell sarcoma; Angiomatoid fibrous histocytoma |
| *EWSR1* | ENSG00000182944 | *DDIT3* | ENSG00000175197 | Myxoid liposarcoma |
| *EWSR1* | ENSG00000182944 | *ERG* | ENSG00000157554 | Ewing sarcoma/PNET |
| *EWSR1* | ENSG00000182944 | *ETV1* | ENSG00000006468 | Ewing sarcoma/PNET |
| *EWSR1* | ENSG00000182944 | *ETV4* | ENSG00000175832 | Ewing sarcoma/PNET |
| *EWSR1* | ENSG00000182944 | *FLI1* | ENSG00000151702 | Ewing sarcoma/PNET |
| *EWSR1* | ENSG00000182944 | *FEV* | ENSG00000163497 | Ewing sarcoma/PNET |
| *EWSR1* | ENSG00000182944 | *NR4A3* | ENSG00000119508 | Extraskeletal myxoid chondrosarcoma |
| *EWSR1* | ENSG00000182944 | *PATZ1* | ENSG00000100105 | Ewing sarcoma/PNET |
| *EWSR1* | ENSG00000182944 | *SP3* | ENSG00000172845 | Soft tissue sarcoma; undifferentiated |
| *EWSR1* | ENSG00000182944 | *TEC* | ENSG00000135605 | Extraskeletal myxoid chondrosarcoma |
| *EWSR1* | ENSG00000182944 | *WT1* | ENSG00000184937 | Desmoplastic small round cell tumour |
| *FUS* | ENSG00000089280 | *ATF1* | ENSG00000123268 | Angiomatoid fibrous histocytoma |
| *FUS* | ENSG00000089280 | *CREB3L2* | ENSG00000182158 | Low-grade fibromyxoid sarcoma |
| *FUS* | ENSG00000089280 | *CREB3L1* | ENSG00000157613 | Low-grade fibromyxoid sarcoma |
| *FUS* | ENSG00000089280 | *DDIT3* | ENSG00000175197 | Myxoid liposarcoma |
| *FUS* | ENSG00000089280 | *ERG* | ENSG00000157554 | Ewing sarcoma |
| *FUS* | ENSG00000089280 | *FEV* | ENSG00000163497 | Ewing sarcoma |
| *JAZF1* | ENSG00000153814 | *PHF1* | ENSG00000112511 | Endometrial stromal sarcoma |
| *JAZF1* | ENSG00000153814 | *SUZ12* | ENSG00000178691 | Endometrial stromal sarcoma |
| *PAX3* | ENSG00000135903 | *FOXO1* | ENSG00000150907 | Alveolar rhabdomyosarcoma |
| *PAX3* | ENSG00000135903 | *FOXO4* | ENSG00000184481 | Alveolar rhabdomyosarcoma |
| *PAX3* | ENSG00000135903 | *NCOA1* | ENSG00000084676 | Alveolar rhabdomyosarcoma |
| *PAX3* | ENSG00000135903 | *NCOA2* | ENSG00000140396 | Alveolar and embryonal rhabdomyosarcoma |
| *PAX7* | ENSG00000009709 | *FOXO1* | ENSG00000150907 | Alveolar rhabdomyosarcoma |
| *SS18* | ENSG00000141380 | *SSX1* | ENSG00000126752 | Synovial sarcoma |
| *SS18* | ENSG00000141380 | *SSX2* | ENSG00000187754 | Synovial sarcoma |
| *SS18* | ENSG00000141380 | *SSX4* | ENSG00000204645 | Synovial sarcoma |
| *SS18L1* | ENSG00000184402 | *SSX1* | ENSG00000126752 | Synovial sarcoma |
| *TAF15* | ENSG00000172660 | *NR4A3* | ENSG00000119508 | Extraskeletal myxoid chondrosarcoma |
| *TCF12* | ENSG00000140262 | *NR4A3* | ENSG00000119508 | Extraskeletal myxoid chondrosarcoma |
| *TFG* | ENSG00000114354 | *NR4A3* | ENSG00000119508 | Extraskeletal myxoid chondrosarcoma |
